# Risky sexual behaviours among young adults attending Higher Learning Institutions in Mbeya, Tanzania: implications for STIs and HIV preventive programs

**DOI:** 10.12688/aasopenres.13123.1

**Published:** 2020-08-27

**Authors:** Ruby Doryn Mcharo, Willyhelmina Olomi, Philippe Mayaud, Sia E. Msuya

**Affiliations:** 1 HIV and Reproductive Health, National Institute for Medical Research-Mbeya Medical Research Centre (NIMR-MMRC), Mbeya, Tanzania; 2 Department of Epidemiology & Biostatistics, Institute of Public Health,, Kilimanjaro Christian Medical University College (KCMUCo), Moshi, Tanzania; 3 Faculty of Infectious & Tropical Diseases, London School of Hygiene and Tropical Medicine (LSHTM), London, WC1E 7HT, UK; 4 Department of Community Health, Institute of Public Health,, Kilimanjaro Christian Medical University College (KCMUCo), Moshi, Tanzania; 5 Community Health Department,, KCMC Hospital, Moshi, Tanzania

**Keywords:** Sexually transmitted infections, STIs, HIV, young adults, adolescents, behavior, risk, Higher learning institutions, Universities, Tanzania

## Abstract

**Background: **High-risk sexual behaviours (HRSBs) among young adults are a key risk for Sexually Transmitted Infections (STIs), HIV and unplanned pregnancies. The World Health Organization has identified the 15-24 year age-group as high-risk for STIs. Students at Higher Learning Institutions (HLIs) may be at higher risk because they are free of immediate parental supervision, a transient migratory population, and probably at peak years of sexual activity. Here, we describe risky sexual behaviours and preventive practices among young adults attending HLIs in Mbeya, Tanzania.

**Methods: **Cross-sectional study was conducted from March 2019 to January 2020 among students aged 18-24 years enrolled in HLIs within Mbeya. A self-administered questionnaire was used to collect information on sexual health education, activity, behaviour and STI knowledge.

**Results: **504 students were enrolled; mean age of 21.5 (SD 1.74) years. 377 (74.8%) students were sexually active. Mean age of first sexual encounter was 18.4 years and 11.6% reported their sexual debut was <15 years. A higher proportion of male students (59.7%) reported their sexual debut with non-steady partners compared with female students (40.9%). Lack of condom use at sexual debut was reported by 43.3% of sexually active students. Consistent condom use during the past 4-weeks was reported at 23.3% and 16.9% among men and women, respectively. Almost 1 in 10 students reported being forced into having sex by someone they were dating. Sex under the influence of alcohol was reported by 25.5% of the students. Nearly 7 in 10 (77%) students had heard of STIs, but only 15% were aware STIs could be asymptomatic.

**Conclusion: **STI prevention programs need to recognize young adults in HLIs as an at-risk population. HLIs must advocate targeted messages to minimize risks to acquiring STIs, offer counselling and support for those experiencing sexual violence, and promote condom use and safer-sex negotiation skills.

## Introduction

In sub-Saharan Africa (SSA), young people comprise almost a quarter of the population and this number is expected to significantly increase by 2050 ^1^. The terms “ *young people*” and “ *young adult*” have been used interchangeably to describe persons within the 10–24 years age category. Adolescents and young adults experience a distinct “ *transition phase*” whereby the family or society does not quite define them as children or yet adults ^2^; and during this phase the habitual position of the parents gets overridden slowly by peers/friends of the same age. During this phase of transition to self-realisation, young adults also begin to experience sexual activities, heightened sexuality curiosities and become prone to preventable sexual health threats ^2, 3^.

Reports have shown young adults are practicing High Risk Sexual Behaviours (HRSBs), but do not seriously perceive a threat to their health as a result of HRSBs. The HRSBs reported among young adults includes: unprotected sex, multiple sexual partners, frequent sexual partner change, transactional sex, alcohol and other substance abuse, sex under the influence of alcohol, and forced sexual encounters in some instances ^4– 7^. Risk perception may be low despite practising HRSBs because of having poor sexual health information ^7, 8^ and limited access to sexual and reproductive health (SRH) care ^2, 7, 9– 11^. The short and long-term consequences of HRSBs among young adults includes: unwanted pregnancies, increased risk of unsafe abortions, increased risk for Sexually Transmitted Infections (STIs) which are largely asymptomatic with long-term effects, and increased risk of getting HIV ^2, 7^.

Students in Higher Learning Institutions (HLIs; Colleges and Universities) might be at a higher risk of HRSBs than other young adults. Campus life is a time " *free*" of immediate parental watch or teacher supervision that may be a case for secondary school adolescents. Apart from being free from parents/teachers, many HLIs have access to student loans and/or grants that makes them financially independent from guardians and parents. At this stage in HLIs, students are trying to establish independence and identity, encounter novel situations ^10^, and are highly experimental with sex, alcohol and other substances. University students are at the age that substance use is said to be at peak, between 18 and 25 years ^12, 13^, and behaviours learnt earlier on life have been shown to affect adult behaviour. Alcohol use and other substance abuse may be influenced by older peers who have already established such behaviour ^13, 14^, or in transactional/cross-generational relationships, these are the older partners and in most cases men. Students in their first years of study are highly at risk of over-drinking as they might not have had the freedom to do so when under immediate parental supervision and at this stage be victims of “ *peer pressure*” in an attempt to blend in and gain recognition ^13– 15^.

HRSBs have been reported among different studies of university students ^12– 14, 16– 20^. Prevalence of HRSBs reported among HLIs students in SSA ranged from 40% to 75.2% for multiple sexual partners, 29.2% to 62% for unprotected sex, with sexual activity at 58.5% ^21, 22^ as compared to North America, China and Europe with sexual activity prevalence of 10% to 15% ^23, 24^. The occurrence of STIs and unwanted pregnancies is higher among young adults in SSA than in other settings ^7^.

Despite the higher occurrence of HRSBs and other negative outcomes among students in HLIs in many SSA countries, several studies on sexual practices among young adults still show low levels of necessary preventive behaviours, such as consistent condom use, delayed sexual debut, reducing the number of sexual partners, testing for STIs/HIV, and abstaining from substance abuse ^12, 13, 18– 20^. In Tanzania, there is limited information on sexual and preventive behaviours among HLIs, their risk perception, knowledge on the consequence of HRSBs and care seeking behaviours.

The Mbeya region, in the Southern highlands of Tanzania, exhibits characteristics of a “ *border town*” with an increased risk for STIs/HIV, and is among the regions with the highest HIV prevalence in Tanzania ^25^. The region also has a substantial number of HLIs. HLIs form platforms of interaction for people from all corners of the country. Universities and colleges relate and interact with their surrounding communities diversely through commercial, as well as sexual, activities. Sexual relationships with members of the nearby communities are preferred for several reasons; yet, little is understood on the HIV/STIs situation, risk, burden and sexual practices among the university students in Mbeya considering the high recorded HIV prevalence of the surrounding community. We describe here the existing risky sexual behaviours and preventive practices among young adults attending HLIs in Mbeya region, Tanzania.

## Methods

### Study design and site

This study followed a cross-sectional design and was conducted from March 2019 to January 2020 in Mbeya region, Tanzania. Mbeya region is located in the the South-West of Tanzania, within the Southern Highlands Zone and it borders, internationally, Zambia and Malawi. The region comprises of a youthful population, and its main economic activities include small-scale agriculture, mining trade and tourism ^26^. There are six HLIs registered by the Tanzania Commission for Universities (TCU) in Mbeya region. These include: Mbeya University of Science and Technology (MUST), Mzumbe University – Mbeya University College (MUMCo), Tanzania Institute of Accountancy (TIA), Mbeya Teofilo Kisanji University (TEKU), Open University of Tanzania (OUT), and St. Augustine University of Tanzania (SAUT).

### Population, sample size and sampling

Eligible participants included Tanzanian students enrolled in HLIs within Mbeya region in any year of study, aged 18–24 years of age and who agreed to provide written informed consent prior to all study-related procedures. Participants were ineligible to take part if they were students attending short-term courses (<6 months) or elective students.

Sample size was determined based on proportions assessment in cross-sectional studies using random sampling and adjusted for non-response, and the minimum sample required was 494.

***Sampling.*** Each HLI in Mbeya region was invited to participate and all six of the HLIs agreed to take part; however OUT did not have any students that fit the age eligibility criteria. A complete electronic list of all students registered, aged 18–24 years of age, was obtained from the Academic Registrars' offices. Based on the varying number of students from each HLI, and the study desired sample size, probability proportional to size was used to determine the total number of students by gender from each HLI. Students were selected using a computerized random number, and not based on the department or course to which they were registered. Each selected student was notified via phone that he/she has been randomly selected to take part in the study and if he/she was willing to take part, he/she was then requested to report to the data collection point within their respective campuses. If the phone number was not reachable or the student selected had no mobile phone, the course representative assisted in physically finding the selected student and a face to face appointment was then scheduled with the student. Each selected student was required to present a student identification proof before study procedures could commence.

### Data collection methods, tools and study procedures

An individual self-administered questionnaire was used in the study ^27^. The questionnaire was administered using a tablet or smart phone through a web-based software ( Open Data Kit; ODK) or hard copy, which ever method the participant preferred. The questionnaire was adopted from various survey questionnaires used in research to assess sexual risk behaviours ( https://www.natsal.ac.uk/media/2097/final-questionnaire_technical-report-appendix-b.pdf; ^20^) and modified to relate to the Tanzanian university setting. The questionnaire was pre-tested on few HLI students from a nearby region (Iringa) before the start of data collection; and any issues that were raised from the pre-testing were factored in to revise the questionnaire in a more practical manner. The questionnaire collected socio-demographic information, sexual health education, knowledge and attitudes on STIs and sexual activity and behaviour.

***Definition of variables.*** There were 33 questions on knowledge and attitudes on STIs and each correct response was given a single mark. Knowledge on STIs was categorized to Good, Moderate or Poor based on correct response above 75%, 45–75% and below 45%, respectively.

### Data management and statistical analysis

The web-based software (ODK) was designed with smart checks for incomplete or ambiguous responses, and responses through the hard copy questionnaire were reviewed for completeness at the end of each day by the Research Assistant. Data was retrieved in an Excel format database, and thereafter cleaned and analysed using statistical software Stata version 14 for Windows ( *Statacorp, College Station, TX 77845, USA*). Data was summarised descriptively using percentages and/or proportions for categorical variables, mean and respective measure of dispersion for numerical variables.

### Ethics

Ethical approval was granted by the Mbeya Medical Research and Ethics Review Committee (MMREC) (Reference Number SZEC-2439/R.A/V.1/07), Kilimanjaro Christian Medical College Research Ethics and Review Committee (CRERC) (Reference Number 2405), and Tanzanian National Health Research Ethics Committee (NatHREC) (Reference Number NIMR/HQ/R.8a/Vol.IX/3092).

All eligible participants who agreed to participate in this study received study information and provided written informed consent to participate prior to any study related activity. Only participant’s study identification number was used in the questionnaire, and only the Principal Investigator and Research Assistant had access to information linking the study identification number and participant’s identifier, for follow-up of results if need be. All results were communicated back to the participants privately as soon as they were confirmed by the study team, and those reporting HRSBs were counselled further on their risk and preventive measures for STIs.

## Results

### Response

Out of a total of 632 students who were sampled randomly and contacted from the HLIs, 504 students aged 18–24 years attending HLIs in Mbeya region were enrolled. Of the 128 who were not enrolled, 32 (25%) were not found and the remaining said they were not willing to participate in the study.

### Characteristics of the participants

The participants’ socio-demographic characteristics are shown in Table 1. The mean age of the 504 students was 21.5 years (SD 1.74). Over 90% were single, and a larger proportion depended on their parent/guardian for financial support. Almost 70% were not permanent residents of Mbeya region.

**Table 1.** Sociodemographic characteristics of the participants (N=504).

Variable	n (%)
Gender - Female - Male	217 (43.1) 287 (56.9)
Religion - Christian - Muslim - Not recorded	427 (84.7) 63 (12.5) 14 (2.8)
Marital status - Single - Married - In a relationship/cohabiting	473 (93.9) 12 (2.4) 19 (3.8)
University year - First - Second - Third - Fourth	223 (44.3) 170 (33.7) 108 (21.4) 3 (0.6)
Employment status - Un-employed - Employed	497 (98.6) 7 (1.4)
Financial source - Parent/Guardian - Sponsorship/Well-wisher - Self/Others	435 (86.3) 37 (7.3) 32 (6.4)
Resident of Mbeya - No - Yes - Not reported	352 (69.8) 151 (30.0) 1 (0.2)

### Behaviour at sexual debut

A total of 377 out of 504 students (74.8%) reported to be sexually active. Out of the 377 sexually active students, their reported behaviour at onset of sexual debut are shown in Table 2. The mean age at first sexual debut was 18.4 years, and this was higher for female (19 years) than male students (17.9 years). Of the 377 students, 11.6% had their sexual debut at ≤15 years. Consensual sex was higher in male (66.4%) compared with female students (35.7%). A higher proportion of male participants (59.7%) reported their sexual debut with a non-steady partner, either someone they did not know well (10.8%), had recently met (10.3%) or had known each other for a while but not in a steady relationship (38.6%); while most female participants reported sexual debut with a partner in a steady relationship (51.3%). Lack of condom or contraceptive use at sexual debut was reported by 43.3% of the 377 students, with no difference between men (43.2%) and women (43.3%). Sexual debut was reported with either a neighbour (25.2%) or a partner met in school or HLI (54.9%). About 6% of the students reported being forced into having sex at debut (9.1% female and 4.0% male students).

**Table 2.** Reported sexual behaviours at sexual debut among participants (N=377).

Variable	Men (N=223) n (%)	Women (N=154) n (%)	Total n (%)
How old were you when you first had sexual intercourse with someone of the opposite sex?			
- <12 - 12–15 - 16–18 - 18 Mean (SD) age of debut	2 (0.9) 37 (16.6) 90 (40.4) 94 (42.1) 17.9 (2.5)	0 (0.0) 5 (3.3) 61 (39.6) 88 (57.1) 19 (1.8)	2 (0.5) 42 (11.1) 151 (40.1) 182 (48.3) 18.4 (2.3)
Who was more willing to have sexual intercourse that first time?			
- Both were equally willing - I was more willing - Partner was more willing - I was forced - Not reported	148 (66.4) 55 (24.7) 10 (4.5) 9 (4.0) 1 (0.4)	55 (35.7) 7 (4.6) 77 (50.0) 14 (9.1) 1 (0.6)	203 (53.9) 62 (16.4) 87 (23.1) 23 (6.1) 2 (0.5)
Which one of these descriptions best applies to you and this person at that time?			
- It was someone I didn’t know - We had recently met - We had known each other for a while, but were not in a steady relationship - We were in a steady relationship - We were living together as a couple/married at the time - Other - Not reported	24 (10.8) 23 (10.3) 86 (38.6) 77 (34.5) 11 (4.9) 2 (0.9) 0 (0.0)	8 (5.2) 11 (7.1) 44 (28.6) 79 (51.3) 7 (4.5) 3 (2.0) 2 (1.3)	32 8.5) 34 (9.0) 130 (34.5) 156 (41.4) 18 (4.8) 5 (1.3) 2 (0.5)
Was any form of contraception used on that occasion? *(multiple responses apply)*			
- Condom - Pills/Injectables - Emergency contraception - No contraception used - Other	119 (52.0) 5 (2.2) 5 (2.2) 99 (43.2) 1 (0.4)	76 (48.4) 5 (3.2) 3 (1.9) 68 (43.3) 5 (3.2)	195 (51.7) 10 (2.7) 8 (2.1) 167 (43.3) 6 (1.6)
How did you meet the person who you first had sexual intercourse with?			
- Neighbour - Introduced by friends/family - At school /university/college - At work - Through sports club/society - On holiday/travel - At a bar/night club - Public space - Other - Not reported	67 (30.0) 10 (4.5) 126 (56.5) 2 (0.9) 4 (1.8) 6 (2.7) 2 (0.9) 4 (1.7) 1 (0.5 1 (0.5)	28 (18.2) 15 (9.7) 81 (52.6) 2 (1.3) 5 (3.3) 9 (5.8) 1 (0.7) 7 (4.5) 6 (3.9) 0	95 (25.2) 25 (6.6) 207 (54.9) 4 (1.0) 9 (2.4) 15 (4.0) 3 (0.8) 11 (3.0) 7 (1.8) 1 (0.3)
Looking back now to the first time you had sexual intercourse:			
- should have waited longer before having sex - should not have waited so long - it was the right time - Not reported	106 (47.5) 38 (17.0) 78 (35.0) 1 (0.5)	87 (56.5) 16 (10.4) 49 (31.8) 2 (1.3)	193 (51.2) 54 (14.3) 127 (33.7) 3 (0.8)
How long was it between when you first met your sexual partner and when you first had sex with him/her?			
- 24 hours or less - Between 1 day and 1 week - Between 1 week and 4 weeks - Between 4 weeks and 6 months - Between 6 months and 1 year - More than 1 year	16 (7.2) 28 (12.6) 32 (14.3) 58 (26.0) 38 (17.0) 51 (22.9)	11 (7.1) 7 (4.6) 24 (15.6) 24 (15.6) 33 (21.4) 55 (35.7)	27 (7.2) 35 (9.3) 56 (14.8) 82 (21.8) 71 (18.8) 106 (28.1)

### Current sexual behaviour among HLIs students

As shown in Table 3, consistent condom use in the past 4 weeks was low, reported only by 23.3% and 16.9% among men and women, respectively. Female students tend to have multiple partners who are five or more years older than them as compared to male students who tend to have multiple partners five or more years younger. In total 20.4% of the 377 students reported being forced into having sex by someone; this was higher in female students (26.0%) than male students (16.6%). Almost 1 in 10 students were forced by someone they were dating. Sex under the influence of alcohol (men, 22.2%; women, 31.6%) and/or illegal drugs (men, 6.7%; women, 3.9%) was among the HRSBs reported. About 12.7% of the students reported being sexually attracted to both sexes (bisexual) and 2.4% being attracted to the same sex (homosexual).

**Table 3.** Reported current risky sexual behaviours among sexually active participants (N=377).

Variable	Men (N=223) n (%)	Women (N=154) n (%)	Total n (%)
Did you use a condom/was a condom used when you had sexual intercourse in the last 4 weeks?			
- Yes, used every time - Yes, used sometimes - No, not used in the last 4 weeks	52 (23.3) 44 (19.7) 127 (57.0)	26 (16.9) 39 (25.3) 89 (57.8)	78 (20.7) 83 (22.0) 216 (57.3)
Past 4 weeks, how many partners were five or more years older than you?			
- 0 - 1 - 2 - ≥3 - Not reported Median (IQR)	176 (78.9) 22 (9.9) 15 (6.7) 9 (4.0) 1 (0.5) 0 (0)	82 (53.3) 39 (25.3) 11 (7.1) 19 (12.3) 3 (2.0) 0 (0-1)	258 (68.4) 61 (16.2) 26 (6.9) 28 (7.4) 4 (1.1) 0 (0-1)
Past 4 weeks, how many partners were five or more years younger than you?			
- 0 - 1 - 2 - ≥3 - Not reported Median (IQR)	157 (70.4) 35 (15.7) 11 (4.9) 13 (5.9) 7 (3.1) 0 (0-1)	121 (78.6) 10 (6.5) 5 (3.3) 9 (5.8) 9 (5.8) 0 (0)	278 (73.8) 45 (11.9) 16 (4.2) 22 (5.9) 16 (4.2) 0 (0)
Have you ever been physically forced to have sexual intercourse when you did not want to?			
- Yes - No - Not reported	37 (16.6) 185 (83.0) 1 (0.4)	40 (26.0) 114 (74.0) 0 (0.0)	77 (20.4) 299 (79.3) 1 (0.3)
During the past 6 months, did someone you were dating or going out with force you to do sexual things that you did not want to do?			
- Yes - Never happened - Not reported	22 (9.9) 199 (89.2) 2 (0.9)	16 (10.4) 136 (88.3) 2 (1.3)	38 (10.1) 335 (88.8) 4 (1.1)
How old were you (years) when you had your first drink of alcohol?			
- <12 - 12-15 - 16-18 - >18 Mean (SD)	3 (4.2) 11 (15.3) 22 (30.5) 36 (50.0) 17.9 (3.2)	3 (7.9) 7 (18.4) 6 (15.8) 22 (57.9) 18.4 (3.3)	6 (5.4) 18 (16.4) 28 (25.5) 58 (52.7) 18.0 (3.2)
Have you ever had sexual intercourse while under the influence of alcohol?			
- Yes - No	16 (22.2) 56 (77.8)	12 (31.6) 26 (68.4)	28 (25.5) 82 (74.5)
Do you drink alcohol before having sexual intercourse?			
- Yes - No	15 (20.8) 57 (79.2)	6 (15.8) 32 (84.2	21 (19.1) 89 (80.9)
How old were you when you first tried marijuana/other illegal drugs?			
- <12 - 12-15 - 16-18 - >18	1 (4.4) 2 (8.7) 8 (34.7) 12 (52.2)	0 (0) 1 (33.3) 0 (0) 2 (66.7)	1 (3.9) 3 (11.5) 8 (30.7) 14 (53.9)
Have you ever had sexual intercourse while under the influence of marijuana/other illegal drugs?			
- Yes - No	15 (6.7) 208 (93.3)	6 (3.9) 148 (96.1)	21 (5.6) 356 (94.4)
How old were you when you first performed oral sex on a man/woman?			
- <12 - 12-15 - 16-18 - >18	1 (1.9) 3 (5.6) 17 (32.1) 32 (60.4)	1 (2.3) 0 (0.0) 10 (22.7) 33 (75.0)	2 (2.1) 3 (3.1) 27 (27.8) 65 (67.0)
Do you read/watch pornographic materials?			
- Yes - No	135 (60.5) 88 (39.5)	50 (32.5) 104 (67.5)	185 (49.1) 192 (50.9)
Do you think of yourself as …			
- Heterosexual (attracted to opposite sex only) - Homosexual (attracted to same sex only) - Bisexual (attracted to men and women) - Prefer not to say - Not reported	141 (63.2) 6 (2.7) 20 (9.0) 56 (25.1) 0 (0.0)	73 (47.4) 3 (2.0) 28 (18.2) 49 (31.8) 1 (0.6)	214 (56.8) 9 (2.4) 48 (12.7) 105 (27.8) 1 (0.3)

### Knowledge and attitude on STIs

The reported knowledge and attitudes on STIs are shown on Table 4. In total 77% had ever heard of STIs, and almost 60% learnt about STIs from lessons at school and friends of about their age group. The level of knowledge on STIs was poor (51.4%) among the 504 respondents; 2.4% had excellent knowledge and only 15% were aware STIs can be asymptomatic. Over three-quarters (69%) of the respondents were concerned and worried about getting an STI as a consequence of unprotected sex than getting unwanted/unplanned pregnancies (43%). Over 90% of the participants felt it is necessary for academic institutions to discuss issues regarding the prevention of STIs, and 69% agreed that screening for STIs is good.

**Table 4.** Knowledge and attitudes on STIs and HIV among HLI students (N=504).

Variable	n(%)
Ever heard of STIs	
- Yes - No	389(77.2) 115(22.8)
How participant learnt about STIs	
- Family members (father/mother/brother(s) or sister(s) - Friends of about my own age - First (girlfriend/boyfriend) or sexual partner - Lessons at school - Media/Books/newspapers/Internet/pornographic websites - Doctor, nurse or clinic - Other	100(19.8) 58(11.5) 3(0.6) 242(48.0) 64(12.7) 32(6.4) 5(1.0)
Level at which Sexual and Reproductive health Education was received ( *multiple* *responses apply*)	
- Primary school - Secondary school O’ level - Secondary school A’ level - University/HLI	67 (13.3) 353 (70.0) 101 (20.0) 266 (52.8)
Level of STI knowledge	
- Poor - Moderate - Excellent	259 (51.4) 233 (46.2) 12 (2.4)
Does using condoms decrease the risk of being infected with an STI?	
- Yes - No - Do not know - Not reported	441(87.5) 43(8.5) 17(3.4) 3(0.6)
Does having multiple sexual partners increase chances of being infected with an STI?	
- Yes - No - Do not know - Not reported	440(87.3) 35(6.9) 24(4.8) 5(1.0)
Do you think people infected with one STI can be at an increased risk of getting HIV?	
- Yes - No - Do not know - Not reported	367(72.8) 81(16.1) 54(10.7) 2(0.4)
Does alcohol intake increase an individual’s susceptibility to STIs?	
- Yes - No - Do not know - Not reported	304(60.3) 127(25.2) 71(14.1) 2(0.4)
Can intake of some illegal drugs increase an individual’s susceptibility to STIs?	
- Yes - No - Do not know - Missing	335(66.5) 90(17.9) 71(14.1) 8(1.6)
Can people with STIs have no symptoms?	
- Yes - No - Do not know - missing	76(15.1) 385(76.4) 37(7.3) 6(1.2)
If you were diagnosed with an STI, would you inform your sexual partner and allow her/him to also receive treatment?	
- Yes - No - Do not know	439(87.1) 31(6.2) 34(6.8)
I feel it is necessary for academic institutions to discuss issues regarding prevention of STIs	
- Agree - Disagree - No comment - missing	461(91.7) 23(4.6) 17(3.4) 3(0.6)
I am worried about contracting STIs	
- Agree - Disagree - No comment - missing	316(63.2) 67(13.4) 115(23.0) 6(1.2)
As an outcome of a sexual encounter, what do you fear most? *(multiple responses* *apply)*	
- Pregnancy - Contractingan STI - Contracting HIV	217(43.1) 231(45.8) 345(68.5)

## Discussion

Findings from this study among students attending HLIs in Mbeya showed that nearly one in ten students had their sexual debut at or below 15 years and nearly half (43.3%) did not use any protection for STIs/HIV or pregnancy at sexual debut. Men were more likely to report sexual debut with a non-steady partner or someone they did not know well. HLIs students also reported low condom use in the past 4 weeks (42.7%) with no difference between men and women. Female students tended to have multiple partners who are older as compared to male students who tended to have younger sexual partners. Forced sexual intercourse was reported; and almost 1 in 10 students were forced by someone they were dating. Other HRSBs reported included sex under the influence of alcohol and/or illegal drugs. Knowledge about STIs was poor (51.4%), and majority (85%) did not know STIs can be asymptomatic.

There have been reports of women initiating sex earlier than their male age-mates ^25^; this was not a finding in this study, where men reported earlier sexual debut than women. The mean age of sexual debut was 18.4 years supporting findings from other studies in Tanzania and studies conducted among University students in Eastern and Western Africa that have shown that the age at sexual debut has increased over time in SSA ^18– 20, 25, 28, 29^. Being in a position to attain higher level education may be protective in a way that these students have been exposed to a number of SRH classes on the importance of delaying sex and unwanted pregnancies during secondary school years, but also being under parent/teacher supervision they have restricted time for sexual relations. Could the possibility of achieving higher level education be looked at as a “ *social vaccine*” that is able to shape SRH decision-making in young adults? If so, HIV and STI prevention programs need to strongly commit to school-going adolescents but also those who are out-of-school as well to reflect on the successes of the school SRH programs.

Sex under the influence of alcohol was a noted risky behaviour in this study, reported by 1 in 4 of the sexually active young people. Judgments to make informed and proper sexual practice choices are often compromised with alcohol and other substance abuse, which become a gateway to risky sexual practices. A state of alcohol intoxication may lead to unsafe sex by exaggerating arousal thoughts and reducing attention to STIs or pregnancy concerns ^13, 15, 30^; and studies have shown alcohol increase vulnerability to sexual coercion and largely contribute to sexual offences in HLIs ^13, 30^. Alcohol drinking and/or substance abuse among young people is influenced by their older peers who have already established such behaviour ^14, 30^, and for the case of HLIs, these could be older students, or in transactional/cross-generational relationships, these are the older partners. Casual or sex with a stranger (also referred to as " *friends with benefits*") is known to occur when under the influence of alcohol following an arranged hook-up by friends/peers ^31^. An Australian study on hangovers and one-night stands among female students significantly associated alcohol consumption with unpleasant sexual experiences and non-condom use ^32^. It is important for HIV and STI preventive programs for young people to note that behaviours learnt early in life affect adult behaviour, thus HRSBs in young adults accumulate a higher STI/HIV risk; and that practices of alcohol drinking as well as other substance abuse are of public health concern.

In this study, over half of the sexually active adolescents do not use condoms regularly. Condoms offer a dual-protective effect to STIs/HIV and unwanted pregnancies, hence low consistent condom use as seen in our findings does increase the risk for contracting STIs/HIV and undesired pregnancies. Research among young people has shown that consistent condom use is not a usual practice and chances of using a condom during a sexual encounter highly depended on whether one's peers were also using condoms ^18– 20, 33^. Condom use can also be seen as a sign of lack of love or trust, so partners especially women, would not like to disappoint their significant others by insisting on condom use ^12^. The study findings do not differ much with studies done among Universities in East Africa that showed 49% and 33% of sexually active students did not use a condom the last time they had sex in Uganda and Kenya, respectively ^18, 19^. It is important to understand further whether the low use of condoms among HLIs students is from lack of access by the young people (issues of cost, lack of access to SRH clinics/outlets), non-provision from the HLI in dorms or campus health facilities, belief of reduced sexual pleasure or other myths and misconceptions. Strengthening promotion of dual prevention of pregnancy and STIs by consistent condom use may improve use. HIV and STI prevention programs must make condom use popular again for young adults; clear messages need to stress on the importance of communicating freely and negotiating sexual matters between sexual partners.

From our findings, one in five sexually active students report being physically forced to have sex when they did not want to, with a higher proportion of women (26%) experiencing this compared with men (16.6%). The use of condoms or other protection is unlikely in this situation, increasing the risk of STIs/HIV and unwanted pregnancies. Studies in Kenya, Uganda and Tanzania, showed that forced sex has been reported by 8.9%, 5% and 14% among University students, respectively; with proportions being higher among female students ^18– 20^. In West Africa, Ouedraogo *et. al* reported the prevalence of forced sex to be 11.9% by a partner ^28^. Students reporting forced sex with a regular partner could probably be those engaging in transactional or cross-generational sex. Transactional sexual relationships involve a richer or resourceful partner with a younger partner, and sometimes differentiating transactional sex and prostitution can be challenging as both practices are non-marital and utilize financial gains in exchange for services, thus increasing vulnerability to STIs and HIV among young people ^34^. These risky sexual practices and acts of sexual violence need to be addressed during STI prevention campaigns. Programs need to empower young people to be able to speak up and report confidently any act of sexual violence without feeling victimized or bearing the blame that it was their own fault. Futher, societies need to discard cultural beliefs or attitudes that support sexual violence.

About one in four students reported having two or more sexual partners, with female students reporting a tendency to have older partners compared to male students who reported having partners who are five years younger. Prevalence of multiple partners in this study is low compared to those reported by Kimiywe *et al.* (50%) in Kenya and Mvungi *et al.* (males, 34.7%; females, 19.3%) in Tanzania ^18, 20^. Sexual relations with partners of a wider age-gap increase the chance of acquiring and further transmitting STIs/HIV. Younger women may engage in sexual relations with older partners to acquire basic needs and economic stability ^18, 19, 34^, and the younger partner in such a relationships has very little, or no, power to negotiate on the status quo of the relationship as compared to the older partner that bears the financial benefits. This increases the STI/HIV risk by having unprotected sex as the older partners prefer younger women believing they are " *clean*" of STIs/HIV and hence would less likely suggest condom use or protected sex ^34– 36^. It is important to note that times have changed and that young adults are venturing further into their sexual nature and exploring. STI prevention campaigns and programs need to consider all these variants and address them accordingly; silence and/or denial that such practices do not exist will further fuel the increase in risk to STIs/HIV among young people.

From our findings the level of STI knowledge reported was poor, and practices of HRSBs were clearly noted. Eight out of ten had ever heard of STIs; and lessons at school, family members and friends of about similar age with the participants were reported as the common ways of learning about STIs. Despite having been introduced to Sexual and Reproductive health Education (SRE) as early as Primary school level, 76% did not know that someone with an STI can be asymptomatic. Many STIs are asymptomatic but the infection can still be sexually transmitted and spread within the community ^7^, and hence is important to deliver these facts in STI prevention messages through programs for young people. Even though STI screening is important, it is also challenging, and many cases remain undetected; therefore HLIs may offer targeted screening for high-risk groups or students who have encountered a high-risk partner or sexual encounter to reduce the risk of transmission. HIV and STI prevention programs may also factor in youth-friendly SRH facilities within regions where young adults may easily access services on risk-reduction, education and counselling that is specific to their needs. It is important to note that other STIs increase the risk of acquiring HIV infection ^37^ and have serious complications as well; hence a holistic approach to STIs is imperative among young people.

The insufficient level of knowledge reported here is inadequate to protect this population or shape their sexual behaviours, risk perception and influence behaviour modification. Tanzania’s SRE in schools has been criticised as being ineffective in including sensitive sexuality matters, lacking enough resources and students do not appreciate the school as a tutor for sexual health matters and hence opt for other sources (such as peers) ^25, 38, 39^. Young people reported curriculum gaps on sexual decision-making, sexual pleasure, relationships, safer sex and condom use, and masturbation. At HLIs, there is a dearth of SRH initiatives and if present, there are concerns on their quality with health education campaigns being labelled and perceived as “ *boring*”, “ *repetitive*” or “ *normal*”; and senior staff members lack commitment to SRH matters ^20^. It is crucial for STIs and HIV prevention programs to understand other contributing factors to the low level of knowledge on STIs and existence of HRSBs among young adults despite early introduction of SRE from Primary education level. HRSBs seldom occur solo, and some authors have noted that following HRSB practices ,mainly inconsistent condom use, multiple sexual partners and frequent partner change, university students are thought to be at a relatively similar STI/HIV risk as high-risk groups such as commercial sex workers ^40^.

Substance abuse hinders condom use, and is reported to be associated with having more than one sexual partner because of the state of intoxication which impairs judgment ^12, 13, 30^. Research has shown that substance and/or alcohol abuse precedes and is a gateway to risky sexual practices, STIs/HIV and unplanned pregnancies ^10, 30^, making substance/alcohol abuse a public health concern. A number of prevention programs focusing on HIV have achieved much with regards to raising awareness to HIV, but not so much on other STIs which young people are also at risk for. HRSBs have been universally reported among university students and although students in HLIs are believed to have a higher risk to STIs and HIV, compared to the general population, this has not been evidenced within their own community ^18, 20, 41^. Targeted STI prevention programs are likely to be successful towards improving SRH among young adults in HLIs and reducing their risk to STIs.

### Study limitations

Limitations from these findings included inability to analyse further other variables that influence young adults’ sexual behaviour, such as parent relations and “ *peer pressure*”. Due to the sensitive nature of the study subject in an African setting, some of the HRSBs reported might be underestimates because some students may have provided socially desirable responses and not the true practice of their sexual behaviours. There have been reports of “sex-related bias” in studies ^42^ of sexual behaviours, such that self-reported behaviours among men and women differ in what is considered as “sex,” whether non-penetrative or otherwise with men over-reporting and women under-reporting occurrences. In addition, some information may have been biased and underestimated from recalling past experiences, especially those of a sexual nature that were unpleasant.

## Conclusion

HRSBs among young adults in HLIs were observed in Mbeya, including reports on multiple sexual partners, low consistent condom use, forced sex and sex under influence of alcohol, implying an increased risk for STIs/HIV and unwanted pregnancies in this population. There is a need to have clear and targeted messages for addressing HRSBs and ways to minimize their risk so as to protect the greater community. When considering STI prevention among young adults in HLIs in Mbeya, priorities need to factor in education to minimize their reported risk to acquiring STIs, counselling and support for those experiencing sexual violence, promoting condom use and safer sex negotiation skills. HIV and STI prevention programs need to be targeted to recognize young adults in HLIs as key and at-risk populations and put in place interventions tailor fit to their needs and environment within and outside campuses. Sexual behaviour is complex and one’s competence in negotiating sexual matters and practices is dependent and accrued on what is learnt early from adolescence; hence, HIV and STI prevention programs need to factor in the correct preventive, risk-reduction messages from childhood.

## Data availability

### Underlying data

Figshare: Risky sexual behaviours among young adults attending Higher Learning Institutions in Mbeya-Tanzania, https://doi.org/10.6084/m9.figshare.12806948.v2 ^27^.

### Extended data

Figshare: Risky sexual behaviours among young adults attending Higher Learning Institutions in Mbeya-Tanzania, https://doi.org/10.6084/m9.figshare.12806948.v2 ^27^.

This project contains the following extended data:

- Copy of the questionnaire used
- Data variables key

Data are available under the terms of the Creative Commons Zero "No rights reserved" data waiver (CC0 1.0 Public domain dedication).
