# Supplementary material for: Risky sexual behaviours among young adults attending Higher Learning Institutions in Mbeya, Tanzania: implications for STIs and HIV preventive programs
Source: AAS Open Res. 2021 Nov 8;3:41. Originally published 2020 Aug 27. [Version 2] doi: 10.12688/aasopenres.13123.2 (PMC10080207; doi:10.12688/aasopenres.13123.2)
Supplement: Peer review history

# Peer review: Reviewer response for version 2

**DOI:** 10.21956/aasopenres.14452.r29514

**Published:** 2023-04-06

**Authors:** Shervin Assari

**Affiliations:** 1 Department of Family Medicine, Charles R Drew University of Medicine and Science, Los Angeles, CA, USA

The revision is satisfactory and various aspects of the paper are improved. Sex/gender differences using statistical interactions are tested. Results and tables are also enhanced and corrected. The references are up to date and implications are also discussed.  I do not have any further comment and I think this version can be final.

Is the work clearly and accurately presented and does it cite the current literature?

Yes

If applicable, is the statistical analysis and its interpretation appropriate?

Yes

Are all the source data underlying the results available to ensure full reproducibility?

No

Is the study design appropriate and is the work technically sound?

Yes

Are the conclusions drawn adequately supported by the results?

Yes

Are sufficient details of methods and analysis provided to allow replication by others?

Yes

Reviewer Expertise:

race, racism, social epidemiology, social determinants, sex and gender, health behaviors

I confirm that I have read this submission and believe that I have an appropriate level of expertise to confirm that it is of an acceptable scientific standard.

# Peer review: Reviewer response for version 2

**DOI:** 10.21956/aasopenres.14452.r28979

**Published:** 2022-12-13

**Authors:** Aldemir B. Oliveira-Filho

**Affiliations:** 1 Study and Research Group on Vulnerable Populations, Institute of Coastal Studies, Federal University of Pará, Bragança, Brazil

Regarding the 1st version of the manuscript, the authors complied with the requests made. The manuscript "Risky sexual behaviors among young adults attending Higher Learning Institutions in Mbeya, Tanzania: implications for STIs and HIV preventive programs" was well planned and executed, its findings are well described and discussed. The conclusion of this study is coherent, consistent and useful for prevention and health promotion. Congratulations and more success to the authors!

Is the work clearly and accurately presented and does it cite the current literature?

Yes

If applicable, is the statistical analysis and its interpretation appropriate?

Yes

Are all the source data underlying the results available to ensure full reproducibility?

Partly

Is the study design appropriate and is the work technically sound?

Yes

Are the conclusions drawn adequately supported by the results?

Yes

Are sufficient details of methods and analysis provided to allow replication by others?

Partly

Reviewer Expertise:

Epidemiology; Public Health; Sexually Transmitted Infections.

I confirm that I have read this submission and believe that I have an appropriate level of expertise to confirm that it is of an acceptable scientific standard.

# Response

**Published:** 2022-12-21

**Authors:** Ruby Mcharo

**Affiliations:** NIMR-MMRC, Tanzania

We appreciate your thorough and relevant review in order to improve our article. Thank you very much.

# Peer review: Reviewer response for version 1

**DOI:** 10.21956/aasopenres.14223.r28750

**Published:** 2021-07-27

**Authors:** Aldemir B. Oliveira-Filho

**Affiliations:** 1 Study and Research Group on Vulnerable Populations, Institute of Coastal Studies, Federal University of Pará, Bragança, Brazil

Congratulations to the authors for the development of the scientific study. Overall, the manuscript is interesting, useful, organized, and well-written.

 However, authors can still make a small adjustment to improve the manuscript. In the topic "Population, sample size and sampling", the authors must present the details of how the sample size was calculated. How did the authors conclude that the minimum sample was 494? Did the authors use information from other studies? Which are? Was any program used for the calculation? How was the assessment of proportions carried out?

 This is important for transparency and reproducibility of findings.

 After including the information, the manuscript is indicated for indexing.

Is the work clearly and accurately presented and does it cite the current literature?

Yes

If applicable, is the statistical analysis and its interpretation appropriate?

Yes

Are all the source data underlying the results available to ensure full reproducibility?

Partly

Is the study design appropriate and is the work technically sound?

Yes

Are the conclusions drawn adequately supported by the results?

Yes

Are sufficient details of methods and analysis provided to allow replication by others?

Partly

Reviewer Expertise:

Epidemiology; Public Health; Sexually Transmitted Infections.

I confirm that I have read this submission and believe that I have an appropriate level of expertise to confirm that it is of an acceptable scientific standard, however I have significant reservations, as outlined above.

# Peer review: Reviewer response for version 1

**DOI:** 10.21956/aasopenres.14223.r27987

**Published:** 2020-10-22

**Authors:** Susan B. Schaffnit

**Affiliations:** 1 Pennsylvania State University, State College, PA, USA

This study reports findings from a cross-sectional survey on sexual attitudes, knowledge and behavior of university students in Mbeya Tanzania. The paper is mostly clearly written. I think with a few edits, the paper will present a nice description of interesting data. My primary suggestions are to (1) add more context to the introduction about the study site, and (2) clarify the presentation of the results. The latter I think is more necessary in order to proceed with indexing 

**Introduction:**

- You note that *“…* *with sexual activity at 58.5%* *as compared to North America,* *China and Europe with sexual activity prevalence of 10% to 15%.”* Is this true for North America and Europe? Based on the 2018 National College Health Assessment 66% of college [university] students had had sex in the past 12 months. Rates I found for European countries were similar, if not higher. Can you clarify what you mean here?
- You note that Mbeya has the highest prevalence of HIV in Tanzania – please specify what the prevalence is in the text.
- *“* *Sexual relationships with members of the nearby communities are preferred for several reasons*“ Why is this? Please explain. In fact, it would be nice to have a little more detail here about Mbeya that would help explain why the sexual behavior of students here is particularly interesting/important and give information that could help interpret your results.

 

**Results:**

- What does ‘sexually active’ mean (74.8% of students were sexually active) – does that mean they had ever had sex? Or had had sex within a recent time frame? Please define. It is particularly important to know how sexually active is defined when you present information on current sexual behavior (Table 3). Because, for example, if sexually active is defined by *ever* having had sex then the “No, not in the past 4 week” response to “Did you use a condom/was a condom used when you had sexual intercourse in the last 4 weeks?” could mean either “No – I did not use a condom when I had sex in the past 4 weeks” or “No – I did not have sex in the past 4 weeks”. 
- How did people who were sexually active differ from those who were not based on sociodemographic characteristics? Please *either* provide this information in Table 1 *or* given that the rest of the statistics are reported only for sexually active respondents, limit Table 1 to sexually active respondents.
- Please provide information about whether men’s and women’s responses were statistically different using an appropriate bivariate test.
- In all tables some questions presumably only were asked of a sub-sample – could you please specify when this is the case and correct the reported statistics as needed. For example, age at first drinking alcohol and the question “Have you ever had sexual intercourse while under the influence of alcohol?” were presumably only asked to people who had ever drunk alcohol. In this case, reporting that 25.5% of respondents had sex under the influence of alcohol is an inflation of the relevant statistic. Of 377 respondents who were sexually active, 267 respondents hadn’t drunk alcohol ever (is that right? I calculated that like this: 377 total sexually active respondents MINUS 110 sexually active respondents who answered the alcohol questions and thus had ever drunk alcohol = 267 sexually active student who had never drunk alcohol). That means that the proportion of sexually active students who had sex under the influence of alcohol was actually 7.4% (28/377). If I am not correct, can you clarify why the numbers are as they are?
- In contrast when you report the proportion who had sex under the influence of drugs, the denominator does seem to include people who had never used drugs. Is that correct?
- Again, the question about age at performing oral sex presumably only applies to those who have even performed oral sex. Can you please specify this in the table and report the appropriate n? 
- Table 4: given that your previous results are presented by sex of participant, it would be nice to see these data presented as such as well. Again, ideally with a statistical test for difference between male and female responses.

 

**Discussion:**

- That ¼ students had sex under the influence of alcohol is one of the first discussion points but, as mentioned above, is that the correct statistic? If not, please revise.

 

 Minor comment:

- “Over three-quarters (69%) of the respondents were concerned and worried about getting an STI as a consequence of unprotected sex than getting unwanted/unplanned pregnancies (43%).” – the word ‘than’ in this sentence doesn’t make sense. Do you mean “Over three-quarters (69%) of the respondents were concerned and worried about getting an STI as a consequence of unprotected sex while 43% worried about unwanted/unplanned pregnancies.”?

Is the work clearly and accurately presented and does it cite the current literature?

Yes

If applicable, is the statistical analysis and its interpretation appropriate?

Partly

Are all the source data underlying the results available to ensure full reproducibility?

Yes

Is the study design appropriate and is the work technically sound?

Yes

Are the conclusions drawn adequately supported by the results?

Partly

Are sufficient details of methods and analysis provided to allow replication by others?

Partly

Reviewer Expertise:

Early marriage, reproductive health, family demography

I confirm that I have read this submission and believe that I have an appropriate level of expertise to confirm that it is of an acceptable scientific standard, however I have significant reservations, as outlined above.
